# *TaRPM1* Positively Regulates Wheat High-Temperature Seedling-Plant Resistance to *Puccinia striiformis* f. sp. *tritici*

**DOI:** 10.3389/fpls.2019.01679

**Published:** 2020-01-15

**Authors:** Jiahui Wang, Wei Tian, Fei Tao, Jingjing Wang, Hongsheng Shang, Xianming Chen, Xiangming Xu, Xiaoping Hu

**Affiliations:** ^1^ State Key Laboratory of Crop Stress Biology for Arid Areas and College of Plant Protection, Northwest A&F University, Yangling, China; ^2^ Agricultural Research Service, United States Department of Agriculture and Department of Plant Pathology, Washington State University, Pullman, WA, United States; ^3^ Pest & Pathogen Ecology, NIAB East Malling Research, Kent, United Kingdom

**Keywords:** wheat stripe rust, high-temperature seeding plant resistance (HTSP), non-species-specific, virus-induced gene silencing (VIGS), NBS-LRR

## Abstract

RPM1 is a CC-NBS-LRR protein that was first shown to be required for resistance to *Pseudomonas syringae* pv. *maculicola* in *Arabidopsis thaliana*. Our previous study showed that *TaRPM1* gene in wheat was upregulated about six times following infection by *Puccinia striiformis* f. sp. *tritici* (*Pst*) under high temperature, compared with normal temperature. To study the function of *TaRPM1* in wheat high-temperature seedling-plant (HTSP) resistance to *Pst*, the full length of *TaRPM1* was cloned, with three copies each located on chromosomes 1A, 1B, and 1D. Transient expression of the TaRPM1-GFP fusion protein in *Nicotiana benthamiana* indicated that TaRPM1 localizes in the cytoplasm and nucleus. Profiling *TaRPM1* expression indicated that *TaRPM1* transcription was rapidly upregulated upon *Pst* inoculation under high temperature. In addition, *TaRPM1* was induced by exogenous salicylic acid hormone application. Silencing *TaRPM1* in wheat cultivar Xiaoyan 6 (XY 6) resulted in reduced HTSP resistance to *Pst* in terms of reduced number of necrotic cells and increased uredinial length, whereas no obvious phenotypic changes were observed in *TaRPM1*-silenced leaves under normal temperature. Related defense genes *TaPR1* and *TaPR2* were downregulated in *TaRPM1*-silenced plants under high temperature. We conclude that *TaRPM1* is involved in HTSP resistance to *Pst* in XY 6.

## Introduction

Wheat (*Triticum aestivum*) yield can be greatly reduced by stripe rust caused by obligate biotrophic pathogen *Puccinia striiformis* f. sp. *tritici* (*Pst*) (Wellings, 2011; Chen, 2014). Currently, stripe rust control is primarily achieved through the use of resistant cultivars (Chen, 2014) and fungicide sprays (Chen et al., 2013). However, the loss of resistance in wheat cultivars has been happening frequently due to rapid emergence of new virulent races in *Pst* (Chen et al., 2009; Hu et al., 2014).

Wheat high-temperature (HT) resistance to *Pst*, a non-race-specific and durable resistance, can be classified into two types: high-temperature adult-plant (HTAP) and high-temperature seedling-plant (HTSP) resistance (Chen, 2013). HTAP resistance expresses or increases when plants are in the adult stage and weather becomes warm, whereas HTSP resistance expresses when wheat seedlings are temporarily exposed to 20°C for only 24 h at the initial stage of *Pst* incubation (Wang et al., 2017a; Wang et al., 2019). Xiaoyan 6 (XY 6) is a typical example of wheat cultivars with HTSP resistance to *Pst*.

Plants have evolved various defense mechanisms against biotic (e.g., bacterial and fungal pathogens) and abiotic stresses (e.g., high temperature, drought, salt, and heavy metals) (Jones and Dangl, 2006; Bhattarai et al., 2016; Jiang et al., 2018). One efficient mechanism, effector-triggered immunity (ETI), is activated upon recognition of a pathogen avirulence (Avr) gene by a resistance (R) protein, leading to an array of defense responses, including hypersensitive response (HR) (Cui et al., 2015), reactive oxygen species (ROS) bursts, and induction of defense-related genes (Kandoth and Mitchum, 2013; Liang et al., 2014; Gao et al., 2017).

To date, more than 100 R genes against 122 different pathogens have been cloned from numerous plant species such as *Arabidopsis*, tomato, potato, barely, rice, and wheat (Hinsch and Staskawicz, 1996; Anderson et al., 1997; Ellis et al., 1999; Feuillet et al., 2003; Tör et al., 2004; Fu et al., 2009; Zhou et al., 2014; Li N. Y. et al., 2017; Qian et al., 2017; He et al., 2018; Klymiuk et al., 2018). R proteins can be divided into several super-families based on their specific conserved motifs, including nucleotide-binding sites (NBS), leucine-rich repeats (LRR), toll-interleukin-1 receptors (TIR), coiled-coils (CC), transmembrane motifs (TM), and protein kinases (PK) (Li X. et al., 2017). The NBS-LRR resistance genes represent the largest R-gene family, and can be further subdivided into two major subclasses: those having a putative CC domain (CC-NB-LRR) and those having a TIR domain (TIR-NB-LRR) (Ellis and Jones, 1998; Dangl and Jones, 2001). The CC-NB-LRR subclass includes genes such as *RPM1* and *RPS2* of *Arabidopsis*, which could recognize specifically avrB and avrRpt2 effectors, respectively (Gopalan et al., 1996; Warren et al., 1998; Xu et al., 2018). TIR-NB-LRR subclass includes rust R gene *L6* in flax (Lawrence et al., 1995) and downy mildew R-genes *RPP5* (Parker et al., 1997) and *RPP1* (Botella et al., 1998) in *Arabidopsis*. The *Arabidopsis RPM1* gene confers resistance against bacterium *Pseudomonas syringae* expressing either of the Type III effectors AvrRpm1 or AvrB (Mackey et al., 2002). RIN4 (RPM1-interacting protein 4) has been identified as a membrane protein for resistance against *P. syringae via* its interaction with RPM1. AvrB and AvrRpm1, secreted into plant cells by the Type III protein secretion system, induce phosphorylation of RIN4, which is perceived by RPM1 and then serves to activate host resistance responses (Gururani et al., 2012). Therefore, RPM1 “guards” the plant against *P. syringae* by perceiving the Avr-dependent modifications of RIN4 (Dangl and Jones, 2001). We showed that *TaRPM1* gene in XY 6 is upregulated rapidly following infection by *Pst* under high temperature, compared with normal temperature (Tao et al., 2018). Thus, TaRPM1 is associated with HTSP; however, the precise roles played by *TaRPM1* in the HTSP resistance to *Pst* has not been elucidated.

Temperature sensitivity of R genes has been widely reported in numerous plants. For example, tobacco *N*-mediated HR against tobacco mosaic virus is activated at 22°C but not at 30°C (Wang et al., 2009). The tomato *Mi-1* gene against root-knot nematodes is inactive above 28°C (Hwang et al., 2000; Jablonska et al., 2007). The *Arabidopsis RPW8* gene, conferring resistance to powdery mildew, is suppressed above 30°C (Xiao et al., 2003). The defense responses conferred by *Arabidopsis* NB-LRR receptor gene *SNC1* is activated at 22°C, but not at 28°C (Yang and Hua, 2004). *Yr36*, an R gene involved in HTAP, confers resistance to *Pst* at relatively high temperatures (25°C to 35°C) but not at low temperatures (e.g., 15°C) (Fu et al., 2009). Previously, we showed transcriptional factors *TaWRKY70* (Wang et al., 2017a), *TaWRKY62* (Wang et al., 2017b), and receptor like kinase *TaXa21* (Wang et al., 2019) positively regulate HTSP resistance to *Pst*.

In the present study, we identified and cloned a highly upregulated NBS-LRR gene *TaRPM1* from XY 6 infected with *Pst* and subsequently exposed to high temperature for 24 h. Silencing *TaRPM1* in XY 6 impaired HTSP resistance to *Pst* with reduced host defense responses, increased *Pst* growth, and decreased the expression levels of *TaPR1* and *TaPR2*. We thus conclude that *TaRPM1* positively regulates the HTSP resistance to *Pst* through the salicylic acid (SA) signaling pathway.

## Materials And Methods

### Identification and Characterization of *TaRPM1*

To clone the *TaRPM1* gene, full-length primers based on the XY 6 transcriptome sequences (Tao et al., 2018) were designed using Primer 5.0 software (**Table S1**). The PCR products were purified, and cloned into the PMD18-T vector (TaKaRa, Tokyo, Japan) for sequencing. A phylogenetic tree of *TaRPM1* and *RPM1* members in other species were generated by the neighbor-joining method (1,000 bootstrap replicates) using MEGA6.0 software. For confirming the copy number of *TaRPM1* in the wheat genome, nucleotide sequence of *TaRPM1* was aligned with the sequence from the wheat genome database (http://www.wheatgenome.org/). Multiple sequence alignment was performed using DNAMAN6.0 software.

### Plant and Fungal Materials, Inoculations, and Treatments

Wheat cultivar XY 6 and *Pst* race CYR32 were used in this study. The methods of growing wheat seedlings, inoculation, and temperature treatment regimes were the same as those described by Wang et al. (2017a). To analyze the expression of *TaRPM1* under different treatments, leaves were sampled at 0, 48, 96, 192, 194, 198, 204, 216, 240, 264, and 312 hpi with *Pst*. At 192 hpi, some plants were exposed to the HT (20°C) treatment for 24 h, whereas the others remained at the NT (15°C) treatment. Control wheat seedlings were inoculated with sterile distilled water. All samples were immediately flash-frozen in liquid nitrogen and stored at −80°C. In all experiments, there were three biological replicates for each treatment and sampling time combination, and three technical replicates for each sample were conduced to qRT-PCR analysis.

### Hormone Treatments and Tissue-Specific Expression Analysis

For hormone treatments, leaves of 2-week-old wheat seedlings were sprayed with one of the following hormones: 100 µM SA, 100 µM MeJA, 100 µM ET, and 100 µM ABA. Each of the hormones was dissolved in 0.1% (v/v) ethanol (Wang et al., 2017a). The mock wheat seedlings were sprayed with 0.1% (v/v) ethanol. Leaves were sampled at 0, 0.5, 2, 6, 12, and 24 h post-treatment. For tissue-specific expression analysis of *TaRPM1*, roots, stems, and leaves were sampled from 2-week-old wheat seedlings under the NT conditions. Three independent biological replicates were used for each treatment and sampling time combination, and three technical replicates were performed for each sample to qRT-PCR analysis.

### RNA Extraction and qRT-PCR Analysis

Total RNA of sampled leaves was extracted using the SV Total RNA Isolation System (Promega, Madison, WI, USA) according to the manufacturer’s instructions. First-strand cDNA was synthesized using the PrimeScript RT Reaction System (TaKaRa, Tokyo, Japan) according to the manufacturer’s instruction. qRT-PCR was performed using UltraSYBR Mixture (Kangwei, Beijing, China) to quantify *TaRPM1* expression. Based on our previous study (Wang et al., 2014), the wheat *Ta26s* gene (ATP dependent 26s proteasome regulatory subunit) expressed stably among different treatments; therefore, *Ta26s* was used as a reference gene for analyses. Relative expression of *TaRPM1* was analyzed using the comparative 2^–∆∆Ct^ method. In all the experiments, three independent biological replicates and three technical replicates of each biological replicate for each sample were analyzed to ensure reproducibility and reliability.

### BSMV-Mediated TaRPM1 Gene Silencing

To generate the BSMV: TaRPM1-1as and BSMV: TaRPM1-2as recombined plasmids, two specific cDNA fragments of *TaRPM1* with the *Not*I and *Pac*I restriction sites were inserted into the BSMV:r vector, respectively. The wheat phytoene desaturase (*TaPDS*) gene was inserted into the BSMV:r vector as a positive control. Two-week-old wheat seedlings were inoculated with each of the four viruses: BSMV:r, BSMV: TaPDS, BSMV: TaRPM1-1as, and BSMV: TaRPM1-2as following a previously published method (Wang et al., 2017a). Wheat seedlings treated with 1x FES buffer were used as a negative control. After incubation for 24 h in the dark, all wheat plants were placed in a growth chamber at 25 ± 1°C. Once photobleaching was observed in the BSMV: PDS infected leaves, the fourth leaves were inoculated with *Pst* race CYR32 and then maintained at 15 ± 1°C. For estimating the silencing efficiency of *TaRPM1*, leaves infected with *Pst* were sampled at 0, 24, 48, and 120 hpi for qRT-PCR. To confirm the *TaRPM1* silencing efficiency and expression level of PR genes, leaves were harvested at 0, 12, 24, 48, 72, and 120 hptt for RNA extraction and qRT-PCR (HT was applied at 0 hptt). Three independent biological replicates were performed for each treatment and sampling time combination, and three technical replicates for each sample were conducted to qRT-PCR analysis.

### Histological Observations

The sampled wheat leaves were decolorized and stained as previously described (Wang et al., 2007). The stained leaf segments were observed under a microscope for hyphal length, colony linear length, number of haustoria, and uredinial length using DP-BSW software (Olympus, Corp., Tokyo, Japan). Autofluorescence of wheat necrotic cells was observed through epiﬂuorescence microscopy. About 30–50 infection sites were examined from 8–10 randomly selected leaf segments for each treatment at each sampling time point. Ten leaves were selected randomly for each treatment to assess pustules number/leaves area. There were three biological replicates for each treatment at each sampling time point.

### Subcellular Localization Analysis

The pCambia-TaRPM1-GFP fusion protein and pCambia1302-GFP control vector were separately introduced into *Agrobacterium tumefaciens* strain GV3101 through electroporation. Five-week-old tobacco plants were transiently transformed with *A. tumefaciens* GV3101 containing pCambia-TaRPM1-GFP or pCambia1302-GFP constructs and then assessed under a fluorescent microscope. An H_2_B-mcherry recombination plasmid was used as a nuclear location marker.

For Western blotting, th**e** total protein was extracted from 500 mg of *N. benthamiana* leaves carrying pCambia-TaRPM1-GFP and was separated by SDS-PAGE gel. GFP protein was detected with the anti-GFP antibody (Sigma-Aldrich, Shanghai, China).

### Statistical Analysis

Analysis of variance (ANOVA) was conducted using SAS software (SAS Institute Inc., Cary, NC, USA). Individual mean comparisons were based on the least significant difference (LSD) test.

## Results

### Cloning and Characterization of *TaRPM1*

**Figure 1:**
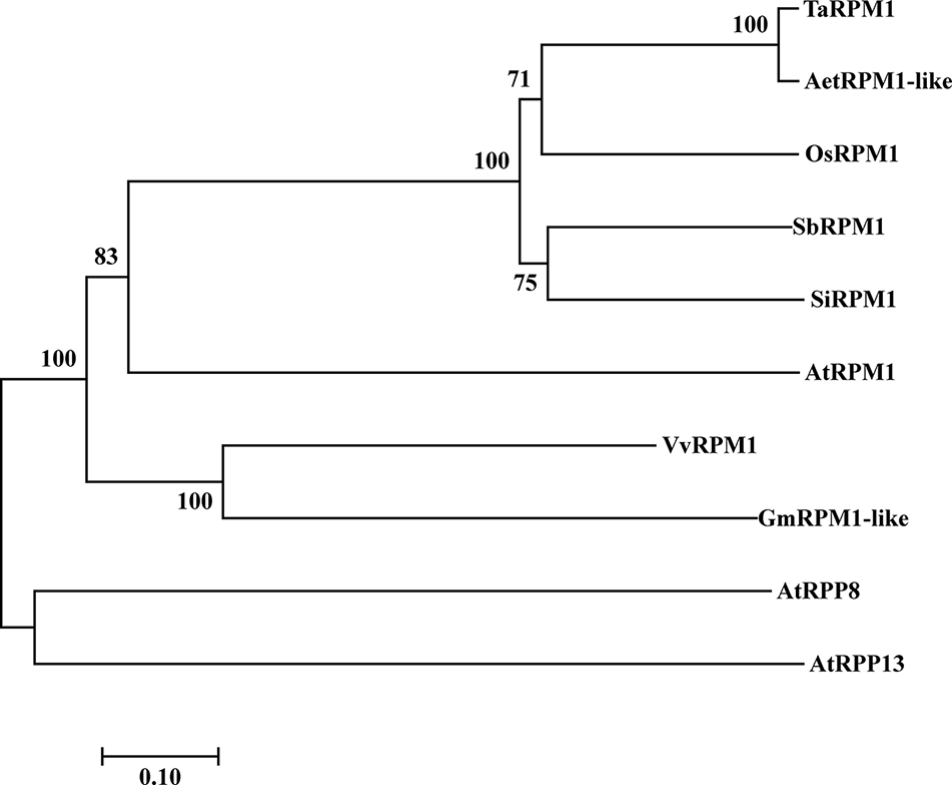
A phylogenetic tree of RPM1 amino acid sequences based on multiple alignments. The GenBank accession numbers of RPM1 protein sequences are as follows: AetRPM1-like (XP_020156667.1), OsRPM1 (XP_015616849.1), SbRPM1 (XP_002449338.1), SiRPM1 (XP_004979032.1), AtRPM1 (AGC12588.1), VvRPM1 (XP_002265617.2), GmRPM1 (XP_003551698.1), AtRPP8 (AAL32592.1), AtRPP13 (AAF42832.1). Aet, *Aegilops tauschii*; Os, *Oryza sativa*; Sb, *Sorghum bicolor*; Si, *Setaria italic*; At, *Arabidopsis thaliana*; Vv, *Vitis vinifera*; Gm, *Glycine max*.

We used the 5’ and 3’ rapid amplification of cDNA ends (RACE) method to clone a 3,325bp cDNA fragment from XY 6, which was selected due to its high expression level of the HTSPresistance after inoculation with *Pst* race CYR32 and subsequently exposed to high temperature for 24 h. The cDNA nucleotide sequence contains an open reading frame (ORF) of 2,754 bp encoding a predicted protein of 917 amino acids with an estimated molecular weight of 103.31 kDa and an isoelectric point value of 6.84. The predicted amino acid sequence of this protein was identical to that of *AetRPM1-like* of *Aegilops tauschii*; thus, this gene is named as *TaRPM1* (GenBank accession number MN647923). Phylogenetic analysis of TaRPM1 and other RPM1 proteins showed that TaRPM1 is most similar to rice OsRPM1 in addition to AetRPM1-like (**Figure 1**). A BlastN search at the International Wheat Genomic Sequence Consortium (IWGSC) revealed that *TaRPM1* shares 94.71% nucleotides with TaRPM1-1AL (**Figure S1**).

### *TaRPM1*Transcript Levels in Different Tissues and Its Response to Hormones

**Figure 2:**
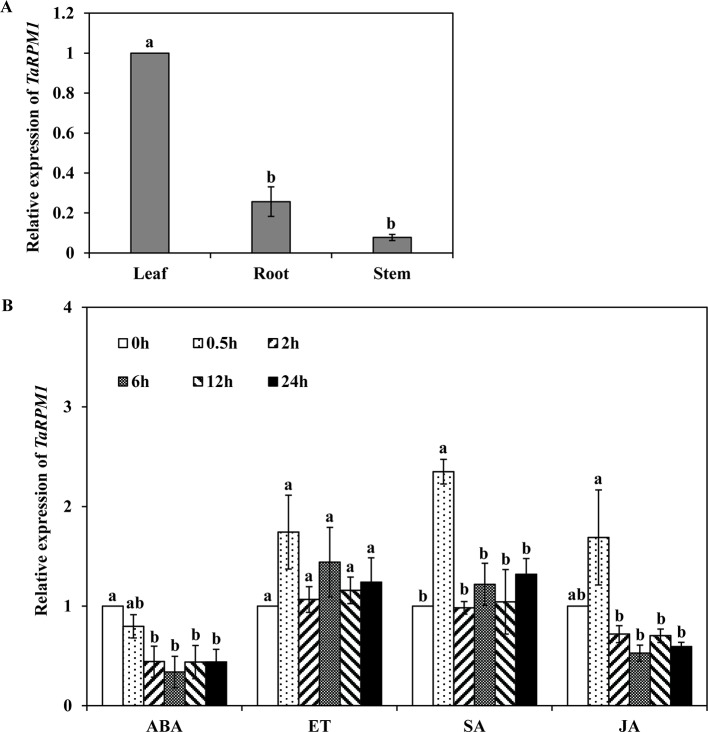
The relative transcript levels of *TaRPM1* in different wheat tissues and in response to different hormones. **(A)** Tissue-specific expression level of *TaRPM1*. **(B)** Responses to hormones: ABA, abscisic acid; ET, ethylene; SA, salicylic acid; MeJA, methyl jasmonate. The mock control was treated with 0.1% (v/v) ethanol. Wheat leaves were sampled at 0, 0.5, 2, 6, 12, and 24 h post–hormone treatment. There were three biological replicates for each treatment and each sampling time point. Three technical replicates for each sample were conducted. Relative transcript levels of *TaRPM1* were calculated using the comparative threshold (2^−ΔΔCT^) method, relative to the mock control at every sampling point. The expression level was standardized as 1 at 0 h. Duncan’s multiple comparison test was conducted to compare between time points for each hormone treatment. The *TaRPM1* expression levels do not differ significantly if they contain at least one common lowercase letter among time points for each hormone treatment.

The relative expression levels of *TaRPM1* in different wheat tissues were determined using quantitative reverse transcription PCR (qRT-PCR). The *TaRPM1* gene was mainly expressed in leaves and, to a less extent, in roots and stems (**Figure 2A**). To study the expression level of *TaRPM1* in response to plant hormones, wheat seedlings were treated with SA, ethylene (ET), methyl jasmonate (MeJA), and abscisic acid (ABA). *TaRPM1* transcription was significantly (*P* < 0.05) increased only upon the SA treatment for 0.5 h. In contrast, when treated with ABA, the expression level of *TaRPM1* was significantly reduced during 2–24 h (**Figure 2B**).

### *TaRPM1* Transcript Level in HTSP Resistance to *Pst*

**Figure 3:**
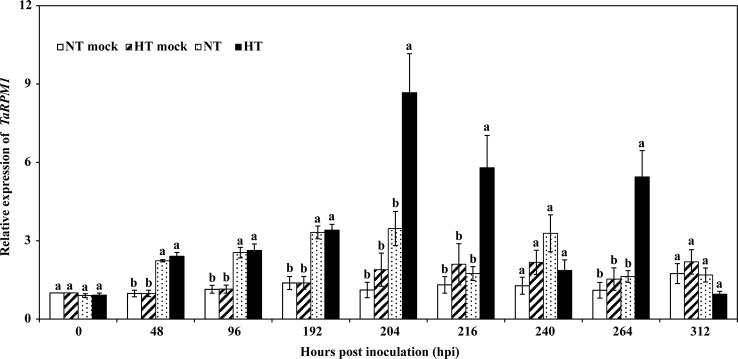
The expression profiles of *TaRPM1* in high-temperature seedling-plant (HTSP) resistance to *Puccinia striiformis* f. sp. *tritici* (*Pst*). NT, wheat leaves were maintained under normal temperature (15°C) after inoculated with *Pst*. HT, wheat leaves were inoculated with *Pst* and transferred to high temperature (20°C) for 24 h at 192 h post-inoculation (hpi). NT mock, non-inoculated wheat leaves were exposed to 15°C. HT mock, non-inoculated wheat leaves were exposed to 20°C for 24 h at 192 hpi. Three independent biological replicates were performed for each treatment and sampling time combination, and three technical replicates for each sample were conducted. The *TaRPM1* expression level in the NT mock leaves at 0 hpi was standardized as 1. Duncan’s multiple comparison test was conducted at the same time point within four different treatments. There are no significant differences at the same time point among the treatments if they share at least one lowercase letter.

To investigate the expression profile of *TaRPM1* in the HTSP resistance to *Pst*, both inoculated and non-inoculated wheat seedlings were subjected to two temperature regimes [high temperature (HT): 20°C for 24 h to induce HTSP, or normal temperature (NT): 15°C] at 192 h post–*Pst* inoculation (hpi), and the leaves were sampled at several time points up to 312 hpi. Compared with the mRNA levels in the mock leaves (non-inoculated leaves), the *TaRPM1* transcript level increased in response to *Pst* inoculation. *TaRPM1* expression from plants exposed to 20°C for 24 h was higher (*P* < 0.05) than that under 15°C at 204, 216, and 264 hpi, and peaked at 204 hpi (**Figure 3**).

### *TaRPM1* Knockdown Compromised Wheat HTSP Resistance to *Pst*

**Figure 4:**
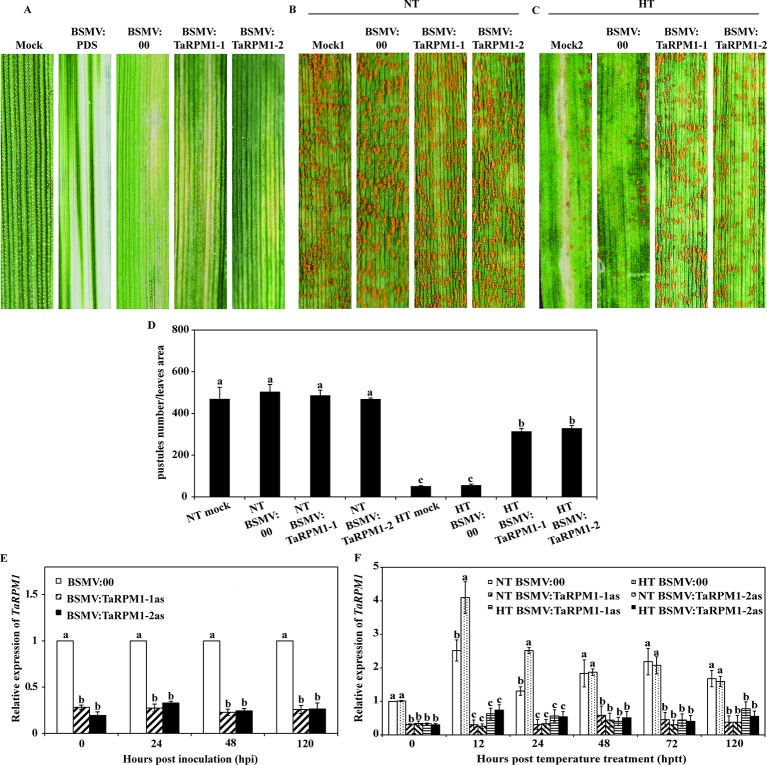
Functional analysis of *TaRPM1* in HTSP resistance to *Pst* using virus-induced gene silencing assay. **(A)** Phenotypic observation on the second leaves inoculated with FES buffer (mock), BSMV: TaPDS, BSMV:00, BSMV: TaRPM1-1as, and BSMV: TaRPM1-2as. The mock leaves and leaves pre-inoculated with BSMV virus were all challenged with *Pst* race CYR32, followed by NT **(B)** and HT **(C)** treatment at 192 hpi. **(D)** Quantification of pustules number in a certain area on different treatment leaves. **(E)** The relative expression levels of *TaRPM1* in silenced and non-silenced leaves infected with CYR32. Leaves were collected at 0, 24, 48, and 120 hpi with CYR32 for RNA extraction and quantitative reverse transcription PCR (qRT-PCR analysis). The *TaRPM1* transcript level in non-silenced leaves at every sampled time point was standardized as 1. **(F)** The relative expression levels of *TaRPM1* in the silenced and non-silenced leaves inoculated with CYR32 under NT and HT treatment. RNA samples were isolated from the leaves first infected with barley stripe mosaic virus (BSMV), and then inoculated with CYR32 at 0, 12, 24, 48, 72, and 120 h post-temperature treatment (hptt) (0 hptt: HT was applied, namely 192 hpi). Three independent biological replicates were performed for each treatment and sampling time combination, and three technical replicates for each sample were conducted. The *TaRPM1* transcript level in NT-treated non-silenced leaves at 0 hptt was standardized as 1. If the treatments share at least one common lowercase letter at the same time point, the *TaRPM1* expression levels do not differ significantly among the these treatments.

To determine the contribution of *TaRPM1* to HTSP in XY 6, we performed barley stripe mosaic virus (BSMV)–based virus-induced gene silencing (VIGS). Two *TaRPM1*-specifc fragments were integrated separately into the BSMV:γ vector to generate BSMV: TaRPM1-1as and BSMV: TaRPM1-2as silencing plants. All of the BSMV-inoculated plants showed stripe mosaic symptoms 10 days after virus inoculation, and the plants inoculated with BSMV: PDS (phytoene desaturase) displayed a photobleaching phenotype 15 days after BSMV inoculation, suggesting the induction of BSMV-mediated silencing (**Figure 4A**). qRT-PCR results indicated that *TaRPM1* silencing efficiency was in the range of 70%–80% (**Figure 4E**). Next, the fourth leaves of XY 6 plants were inoculated with *Pst* race CYR32. Fifteen days after *Pst* inoculation, XY 6 showed a susceptible response under the NT treatment with numerous uredinia on the mock and BSMV:00 leaves (**Figures 4B**, **D**). The non-silenced leaves with a higher expression level of *TaRPM1* showed a resistant response to *Pst* under the HT treatment; and in contrast, the *TaRPM1*-silenced leaves had greater fungal growth than with the non-silenced leaves under HT (**Figures 4C, D, F**).

**Figure 5:**
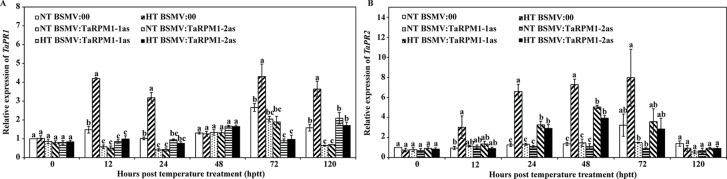
The relative transcript levels of *TaPR1* **(A)** and *TaPR2* **(B)** in *TaRPM1*-silenced plants infected with race CYR32 of *P. striiformis* f. sp. *tritici* exposed to different temperature treatments. Three independent biological replicates were performed for each treatment and sampling time combination, and three technical replicates for each sample were conducted. The expression levels of *TaPR1* and *TaPR2* in the NT-treated leaves pre-inoculated with BSMV:00 at 0 hptt was standardized as 1. There are no significant differences at the same time point among treatments if they share at least one common lowercase letter.

To confirm the host response in *TaRPM1*-silenced plants upon *Pst* infection and the HT treatment, we measured the transcript levels of two pathogenesis-related (PR) protein genes in the SA-mediated signaling pathway. *TaPR1* (**Figure 5A**) and *TaPR2* (**Figure 5B**) were both significantly decreased (*P* < 0.05) in the two HT-treated *TaRPM1*-silenced leaves compared with the HT-treated but non-silenced leaves.

### Histological Observation of *Pst* Growth and Host Response

**Figure 6:**
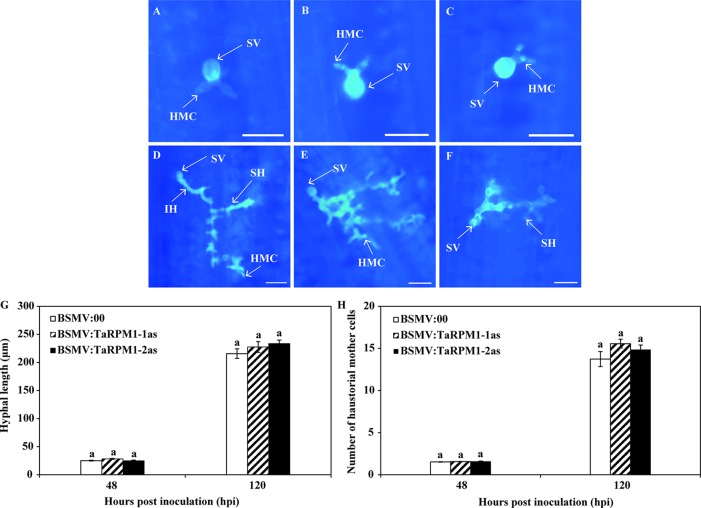
Histological observations of *Pst* development and host responses in *TaRPM1*-silenced leaves under normal temperature. The images of the fourth leaves inoculated with BSMV:00 **(A, D)**, BSMV: TaRPM1-1as **(B, E)**, and BSMV: TaRPM1-2as **(C, F)** were taken under a ﬂuorescence microscope at 48 hpi (**A, B, C,** bar, 50 μm) and 120 hpi (**D, E, F**, bar, 100 μm). SV, substomatal vesicle; HMC, haustorial mother cell; IH, infection hypha; SH, secondary hypha. Hyphal length **(G)** and the number of haustorial mother cells **(H)** were assessed at 48 and 120 hpi using the DP-BSW software after *Pst* were stained. There are no significant differences at the same time point among treatments if they share at least one common lowercase letter. Three independent biological replicates were performed for each treatment and sampling time combination.

We examined leaves microscopically to determine histological changes in *TaRPM1*-silenced leaves at the initial stage of *Pst* development (**Figures 6A**–**F**). At 48 and 120 hpi, hyphal length and number of haustorial mother cells did not differ significantly between BSMV:00 control and *TaRPM1*-silenced plants (**Figures 6G, H**).

**Figure 7:**
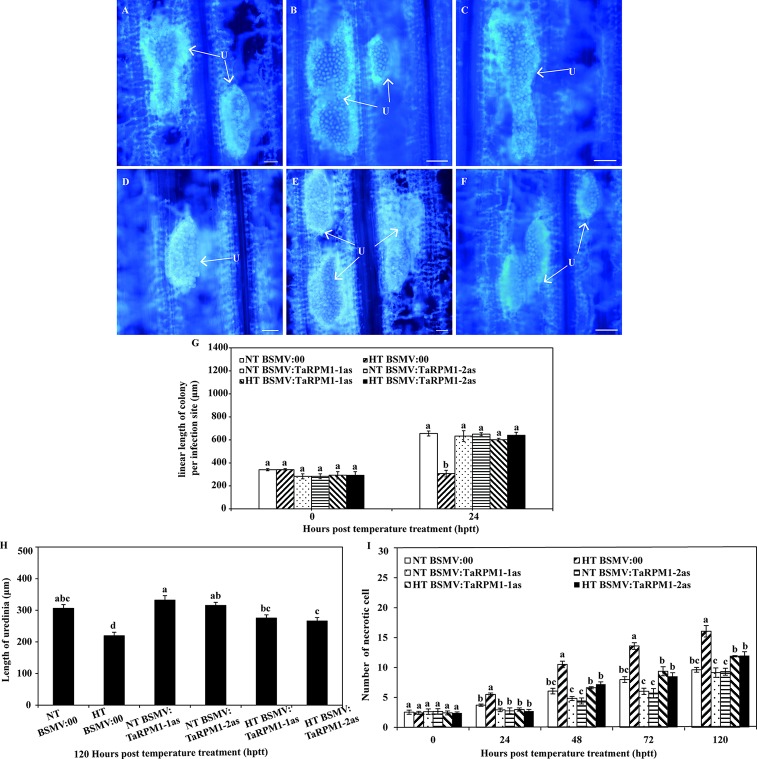
Silencing *TaRPM1* in wheat leaves induces growth of the *Pst* under high temperature. The urediniospores in non-silenced leaves (**A, D,** bar 100 μm) and *TaRPM1*-silenced **(B, C, E, F,** bar, 100 μm) under NT **(A, B, C)** and HT **(D, E, F)** were observed and photographed at 24 hptt. The colony length **(G)** uredinium lengths **(H)** and numbers of necrotic cells **(I)** from 30–50 randomly selected infection sites were calculated. Three independent biological replicates were performed for each treatment and sampling time combination. There are no significant differences at the same time point among treatments if they share at least one common lowercase letter.

At 192 hpi, plants inoculated with *Pst* were subjected to one of the two temperature regimes (HT and NT). For the NT treatment, fusion of urediniospores was observed (**Figures 7A**–**C**), and there were no apparent differences in the uredinium length, the linear length of colony per infection site, and the number of necrotic cells between NT-treated BSMV:00 and NT-treated *TaRPM1*-silenced plants. In contrast, uredinia were sparsely distributed in the HT-treated leaves (**Figures 7D**–**F**), the colony length in the HT-treated TaRPM1-silenced leaves was grater (*P* < 0.05) than that the HT-treated but non-silenced leaves at 24 h post-temperature treatment (hptt) (**Figure 7G**), and similarly the uredinium length in the HT-treated *TaRPM1*-silenced leaves was greater (*P* < 0.05) than that in the HT-treated non-silenced leaves 312 hpi, i.e., 120 h hptt (**Figure 7H**). There were fewer (*P* < 0.05) necrotic cells in the HT-treated TaRPM1-silenced leaves than in the HT-treated non-silenced leaves from 24 to 120 hptt (**Figure 7I**).

### *TaRPM1* Localization

**Figure 8:**
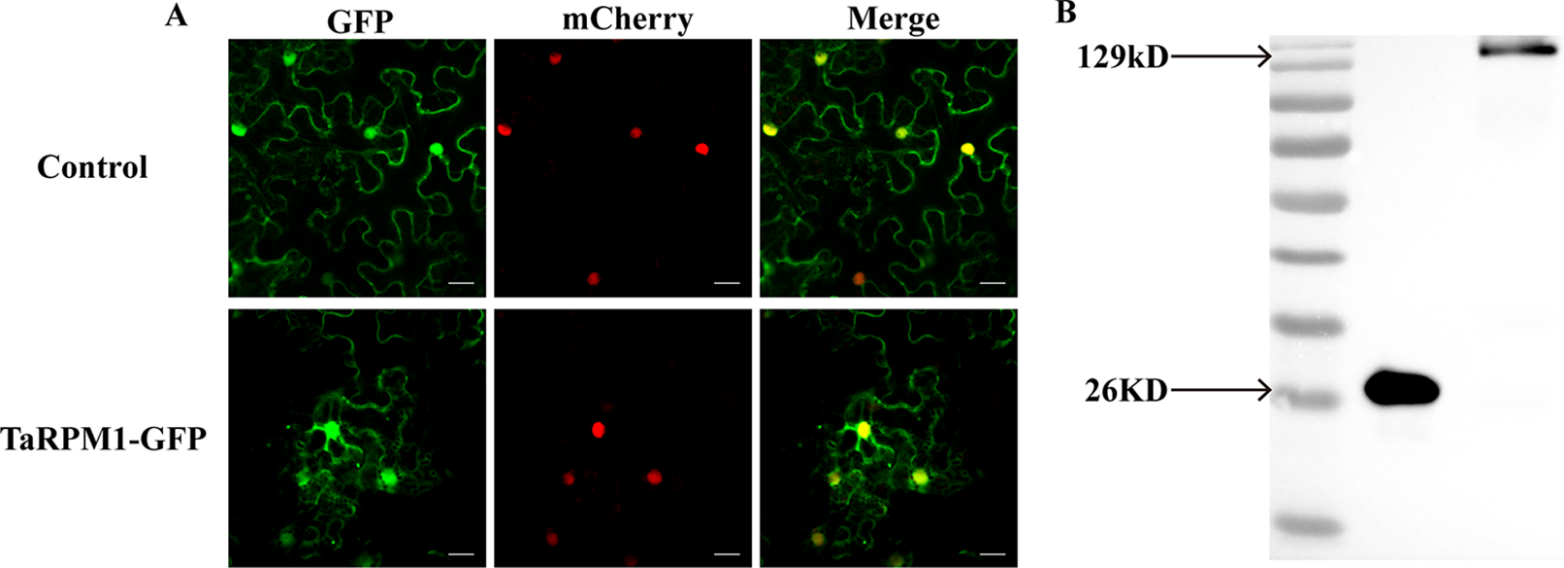
Subcellular localization of protein TaRPM1. **(A)** The TaRPM1-GFP fusion protein and green fluorescent protein (GFP) were separately expressed in *Nicotiana benthamiana*. The histone protein, AtH_2_B, was used as a nuclear location marker gene. Bar, 20 μm. **(B)** Western blot analysis of GFP and TaRPM1-GFP fusion protein.

Prediction of subcellular localization using WoLF PSORT indicated that TaRPM1 was most likely located in the cytoplasm (kNN value, cyto: 4, plas: 4, chlo: 2, E.R.: 2, nucl: 1). To verify this prediction, we produced the fusion construct 35S-TaRPM1-GFP and the control vector 35S-GFP and transformed them into tobacco (*Nicotiana benthamiana*) cells. Fluorescence microscopy showed that the TaRPM1-GFP fusion protein was expressed in both the cytoplasm and the nucleus, similarly to the control vector 35S-GFP (**Figure 8A**). To confirm these results, we further conducted Western blot to analyze the stability of the TaRPM1-GFP fusion protein and found both GFP and the TaRPM1-GFP fusion protein expressed successfully (**Figure 8B**).

## Discussion

NBS-LRR genes, the largest class of R genes, have been reported to play important roles in initiating plant pathogen-triggered immunity (Tameling and Takken, 2008; Gao et al., 2010). As intracellular receptors, NBS-LRR proteins could bind pathogen effectors directly or indirectly through perceiving effector-induced modifications to other proteins, inducing host defense responses (Eitas and Dangl, 2010; Li et al., 2015). To date, numerous NBS-LRR genes have been reported to be involved in resistance to pathogens (Axtell and Staskawicz, 2003; Mackey et al., 2003; Shao et al., 2003; Cherkis et al., 2012; Jun et al., 2016; Macqueen et al., 2016; Zhu et al., 2017). However, functions of NBS-LRR genes in the wheat HTSP to *Pst* remain unknown. In the present study, we demonstrated that *TaRPM1* is a candidate gene required for HTSP resistance to *Pst*. Furthermore, silencing *TaRPM1* also affected *Pst* colony development as well as uredinium length. It may be, therefore, concluded that *TaRPM1* positively contributes to the HTSP resistance to *Pst* in wheat cultivar XY 6.

There are differences in effects of temperature on various R gene–mediated resistance. Many defense responses activated by R genes can be suppressed at higher temperature. For instance, *SNC1* (Yang and Hua, 2004), *N* (Wang et al., 2009), and *RPS4*-mediated resistance are inhibited at 28°C (Gassmann et al., 1999), whereas *Rx* gene against *Potato virus X* is inactive at 30°C (Bendahmane and Baulcombe, 1999). In contrast, *Yr36* could induce HTAP resistance to *Pst* when wheat were grown above 25°C (Fu et al., 2009). Different from these R genes, a short period of exposure 20°C in the early stage of *Pst* development can activate *TaRPM1*-meditated resistance to *Pst*, but not at 15°C. These results showed differences in the temperature sensitivity of R protein activation. Plants with temperature-sensitive R genes may have evolved to balance the energies required for growth and defense when perception of a temperature change (Alcázar and Parker, 2011). For example, the temperature change could result in (i) reorganization of energy resources, which may be followed by reducing available nutrient to rust (Viola and Davies, 1994; Grof et al., 2010), or (ii) production of metabolites that may inhibit the fungal growth (Berger et al., 2007). Thus, we speculate that resistance changes induced by temperature may be through dynamic interactions between plants and the fungal pathogen.

*P. syringae* effectors AvrRpm1 and AvrB induce RIN4 phosphorylation, which is required for activation of RPM1-mediated plant defense responses (Mackey et al., 2002). In contrast to AtRPM1, the interaction between TaRPM1 and TaRIN4 was not detected using the yeast two-hybrid system in the present study (data not shown), indicating that the molecular mechanism of *TaRPM1* involved in the HTSP resistance to *Pst* differs from *AtRPM1*-mediated resistance against *P. syringae*. Further investigation will shed light on whether *TaRPM1* recognizes the effector(s) directly or indirectly with help from other proteins.

NBS-LRR proteins that are involved in the host defense responses may differ in their subcellular locations. RRS1-R (Bernoux et al., 2008), RPS4 (Wirthmueller et al., 2007), MLA10 (Shen et al., 2007), N (Caplan et al., 2008), and Rx1 (Slootweg et al., 2010) are located in the nucleus; host defense responses are critically dependent on the nuclear localization of these NBS-LRR proteins. In addition, plasma-membrane localization of *Arabidopsis thaliana* RPS5 and RPM1 (Boyes et al., 1998; Gao et al., 2011; Qi et al., 2012) is required for their functions. The present study showed that TaRPM1 is located in both nucleus and cytoplasm, differing from *A. thaliana* RPM1. Further research is needed to assess whether TaRPM1 nuclear and/or cytoplasm distribution is of critical importance for its function in the HTSP resistance to *Pst*.

Various host defense responses, including ROS production and programmed cell death, are activated upon perception of pathogens through NBS-LRR proteins (Andersson et al., 2006; Gao et al., 2013; Qi and Innes, 2013). *Arabidopsis* plants with the GhDSC1-overexpressing heterologously display strong resistance to *Verticillium*, with ROS accumulation abundantly and cell death (Li et al., 2019). In our study, silencing *TaRPM1* has no effects on the number of necrotic cells for the NT treatment. In contrast, the number of necrotic cells in the HT-treated TaRPM1-silenced leaves significantly decreased compared with the HT-treated non-silenced leaves, indicating cell death induced by *TaRPM1* was only promoted for the HT treatment. Moreover, number of necrotic cells in the HT-treated TaRPM1-silenced leaves was still more than that in the NT-treated TaRPM1-silenced leaves at 48, 72, and 120 hptt, suggesting that genes other than *TaRPM1* might also be involved in regulating the HTSP to *Pst*.

Several studies have shown that hormones are involved in the NBS-LRR protein-mediated defense responses (Qi and Innes, 2013; Roberts et al., 2013). SA, an important plant defense signaling molecule against biotrophic pathogens, increases transcription of many PR proteins and systemic acquired resistance (SAR) (Liu et al., 2018). Numerous R gene–mediated resistance responses are related to the SA signaling pathway (Shirano et al., 2002). Treatment with SA in *Arabidopsis* leads to increased expression of R genes *RPW8.1* and *RPW8.2*, and resistance to powdery mildew (Xiao et al., 2003). NBS-LRR R gene *TaRGA* is induced significantly by exogenous applications of SA, and silencing *TaRGA* leads to compromised resistance to wheat powdery mildew cause by *Blumeria graminis* f. sp. *tritici* and reduced expression of *PR1* (Wang et al., 2016). In the present study, the highest induction of *TaRPM1* occurred after the SA treatment for 0.5 h, and *TaPR1* and *TaPR2* (two marker genes of the SA pathway) had significantly lower expressions in the HT-treated *TaRPM1*-silenced plants than in non-silenced HT-treated plants, indicating that *TaRPM1* plays a positive role in the HTSP resistance to *Pst* through the SA signaling pathway.

In summary, we demonstrated that NBS-LRR gene *TaRPM1* positively contributes to the HTSP resistance to *Pst* in wheat cultivar XY 6. In addition, the present results suggested that *TaRPM1* confers HTSP resistance to *Pst* though the SA signaling pathway. However, further research is needed to investigate whether *TaRPM1* could recognize effector(s) and to ascertain the exact molecular mechanisms in the TaRPM1-mediated HTSP resistance to *Pst*.

## Data Availability Statement

All datasets generated for this study are included in the article/**Supplementary Material**.

## Author Contributions

XH, HS, XC, and XX planned and designed the research. JiaW, WT, FT, and JinW performed the experiments. XH, JiaW, XC, and XX wrote the manuscript.

## Conflict of Interest

The authors declare that the research was conducted in the absence of any commercial or financial relationships that could be construed as a potential conflict of interest.

## Supplementary Material

Figure S1 Sequence alignment of *TaRPM1* and three copies in the wheat genomes. The blue and yellow boxes represent the primer locations for the VIGS1 and VIGS2 fragments of *TaRPM1*, respectively and the red box represents the primer locations for qRT-PCR of *TaRPM1*.

Table S1 Primers used in this study.
