# Supplementary figures and images for: TaRPM1 Positively Regulates Wheat High-Temperature Seedling-Plant Resistance to Puccinia striiformis f. sp. tritici
Source: Front Plant Sci. 2020 Jan 15;10:1679. doi: 10.3389/fpls.2019.01679 (PMC6974556; doi:10.3389/fpls.2019.01679)

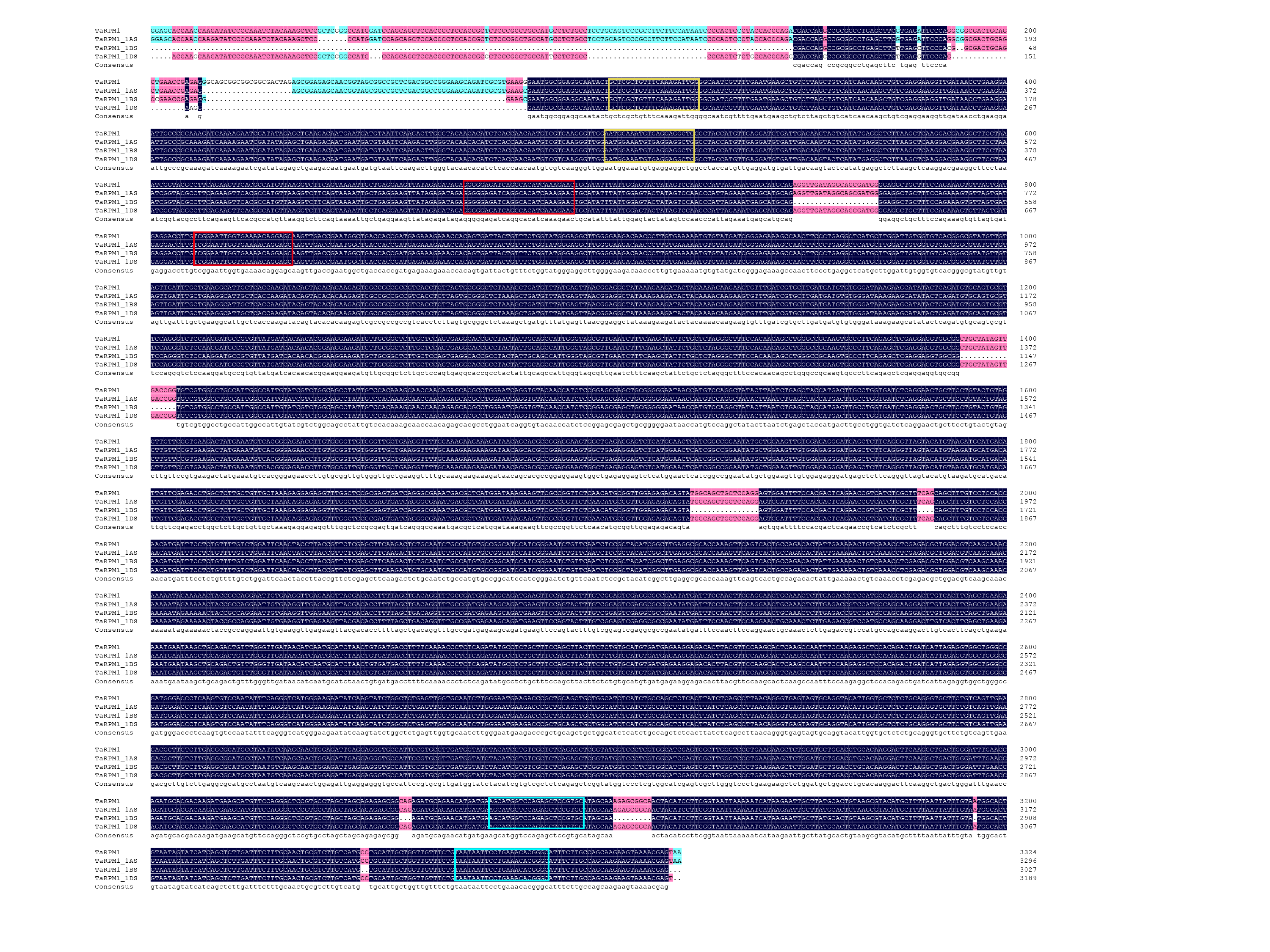

Supplement: Figure S1 — Sequence alignment of TaRPM1 and three copies in the wheat genomes. The blue and yellow boxes represent the primer locations for the VIGS1 and VIGS2 fragments of TaRPM1, respectively and the red box represents the primer locations for qRT-PCR of TaRPM1. [file Image_1.tif]
