# Supplementary material for: TaRPM1 Positively Regulates Wheat High-Temperature Seedling-Plant Resistance to Puccinia striiformis f. sp. tritici
Source: Front Plant Sci. 2020 Jan 15;10:1679. doi: 10.3389/fpls.2019.01679 (PMC6974556; doi:10.3389/fpls.2019.01679)
Supplement: Table S1 — Primers used in this study. [file Table_1.docx]

Table S1. Primers used in this study.

| Primer name | Function | | Primer sequences (5’-3’) |
| --- | --- | --- | --- |
| GSP5 | 5’RACE of *TaRPM1* | | GCTCCTGTTTTCACCAATTCCGA |
| GSP3 | 3’RACE of *TaRPM1* | | GTGGCATCGAGTCGCTTGGGTC |
| *TaRPM1*  *TaRPM1*  *TaRPM1*  *TaRPM1*  *Ta26S*  *TaPR1*  *TaPR2*  TaRPM1*-*GFP | | Full cDNA of *TaRPM1*  qRT-PCR  VIGS vector1 construction  VIGS vector2 construction  qRT-PCR  qRT-PCR  qRT-PCR  Vector construction of  subcellular localization | F:GGAGCACCAACCAAGATATCCCCAA  R:ATTACTCGTTTTACTTCTTGCTGGC  F:GGGGAGATCAGGCACATCAAAGAAC  R:GCTCCTGTTTTCACCAATTCCGA  F:ATATTAATTAAAGCGGCAACTACATCCTTCGGTA  R:TATGCGGCCGCGCCCGTGTTTCAGGAATTATTA  F:ATATTAATTAAGCTCGCTGTTTCAAAGATTGG  R:TATGCGGCCGCCAGCCTCCTCACATTTCCATT  F:GCTGGCTCGTTCAACTGATG  R:GGACCAAGCGTTCTGATTACTC  F:GAGAATGCAGACGCCCAAGC  R:CTGGAGCTTGCAGTCGTTGATC  F:AGGATGTTGCTTCCATGTTTGCCG  R:AAGTAGATGCGCATGCCGTTGATG  F:CCATGGATGGCGGAGGCAATACT  R:ACTAGTGGCACGGAGCCCTGGAACATGCTTC |
